# Patients with symptomatic uncomplicated diverticular disease have high fecal bile acid concentrations

**DOI:** 10.3389/fmed.2025.1533644

**Published:** 2025-07-09

**Authors:** Tsumugi Jono, Yuki Kasai, Takaomi Kessoku, Kosuke Tanaka, Michihiro Iwaki, Takashi Kobayashi, Kota Takahashi, Kosuke Seita, Takayuki Kato, Eiji Sakai, Takeo Kurihashi, Machiko Nakatogawa, Shunsuke Oyamada, Seiji Futagami, Kok-Ann Gwee, Atsushi Nakajima, Antonio Tursi

**Affiliations:** ^1^Department of Gastroenterology and Hepatology, Yokohama City University Graduate School of Medicine, Yokohama, Japan; ^2^Department of Gastroenterology, Yokohama Sakae Kyosai Hospital, Yokohama, Japan; ^3^Department of Gastroenterology, International University of Health and Welfare Graduate School of Medicine, Narita, Japan; ^4^Department of Palliative Medicine and Gastroenterology, International University of Health and Welfare Narita Hospital, Narita, Japan; ^5^Department of Gastroenterology, International University of Health and Welfare Atami Hospital, Atami, Japan; ^6^Department of Internal Medicine, Yokohama Clinic, Kanagawa Dental University, Yokohama, Japan; ^7^Department of Internal Medicine, Namiki Koiso-Medical Clinic, Yokohama, Japan; ^8^Department of Biostatistics, JORTC Data Center, Tokyo, Japan; ^9^Division of Gastroenterology, Nippon Medical School, Tokyo, Japan; ^10^Department of Medicine, Yong Loo Lin School of Medicine, National University of Singapore, Singapore, Singapore; ^11^Territorial Gastroenterology Service, Azienda Sanitaria Locale Barletta-Andria-Trani, Andria, Italy

**Keywords:** diverticular diseases, diverticulosis, bile acid, endotoxins, abdominal pain

## Abstract

**Background and Aim:**

Symptomatic uncomplicated diverticular disease (SUDD) causes persistent pain and impairs patient quality of life; however, its pathogenesis remains unknown. This study investigated the relationship between SUDD and the inflammatory effects of intestinal bile acids (BAs).

**Methods:**

Five institutional cohorts with 361 total patients who received outpatient treatment for abdominal symptoms (from 2020 to 2022) were included in this prospective cohort study. All patients underwent colonoscopy. SUDD was defined as the presence of recurrent abdominal symptoms—pain in the lower quadrant lasting >24 h—in patients with diverticulosis at the site of pain. Patients with diverticula were classified into SUDD and non-SUDD groups. The healthy control (HC) group comprised people with no history of medications and no evidence of colonic diverticula. Liquid chromatography-mass spectrometry determined the concentration of fecal BAs. Fecal calprotectin and blood endotoxin activity assay (EAA) levels were measured.

**Results:**

Total fecal BA concentrations did not differ between HC and non-SUDD patients; however, BA levels were significantly higher in patients with SUDD. Fecal calprotectin and blood EAA levels were significantly higher in the SUDD and non-SUDD groups than in the HC group, and in the SUDD group than in the non-SUDD group. Total BA was mildly positively correlated with fecal calprotectin and blood EAA.

**Conclusion:**

Fecal BA concentrations were significantly increased in patients with SUDD compared with patients without SUDD or healthy subjects, suggesting that fecal BAs might be involved in the pathogenesis of SUDD and that controlling fecal BA levels may be therapeutic for SUDD.

## Introduction

Diverticulosis of the colon is characterized by the presence of diverticula within the colon, and it manifests as a bulging herniation of the mucosa and submucosa. Diverticulosis is the most frequent anatomical alteration in the colon and presents asymptomatically in most cases. Alternately, symptomatic diverticulosis without complications is termed symptomatic uncomplicated diverticular disease (SUDD) and has been reported in 6%–25% patients with diverticulosis in Europe (1). In outpatients with unexplained abdominal symptoms in Asia, the proportion of patients with SUDD was 31% of the total number of patients with diverticulosis (2). As previously reported, SUDD is associated with persistent pain and diarrhea and leads to a lower quality of life (QOL) comparable to that reported for patients with ulcerative colitis or Crohn’s disease (2–5). In addition, 4.3% patients with diverticulosis develop diverticulitis, and 10%–25% patients with SUDD develop diverticulitis (6, 7). Although patients with SUDD require treatment, the pathogenesis of the disease remains unknown, and symptomatic treatment is the mainstay of management (8). Expression levels of the neuropeptide receptor neurokinin 1 and inflammatory cytokine tumor necrosis factor are higher in patients with SUDD than in patients with asymptomatic diverticulosis (9). Additionally, patients with SUDD are characterized by higher levels of fecal calprotectin, which reflects neutrophil infiltration in the intestinal mucosa (10).

Secondary bile acids (BAs) are part of the biosynthesis of primary BAs in the liver and are converted by microorganisms in the intestinal tract, where they exert their inflammatory properties in the colon (11). Alteration of BAs absorption may cause diarrhea (12), and administration of elobixibat, an ileal BA transporter inhibitor, to patients with chronic constipation has been shown to increase total BA concentration in the colon and improve constipation (13). BAs is often involved in the pathophysiology of chronic diarrhea, arising from organic disease such as Crohn’s disease (14) or from functional disease such as Irritable Bowel Syndrome (IBS) (15). Since diarrhea may occurs also in SUDD (2, 16), we hypothesized that BAs may be involved in the pathogenesis of SUDD, at least in causing diarrhea. Thus, the aim of this study was to determine the relationship between SUDD and BA levels.

## 2 Materials and methods

### 2.1 Study participants

This prospective cohort study included five institutional cohorts of patients referred to outpatient clinics (Yokohama City University Hospital, Kanagawa Dental University Yokohama Clinic, International University of Health and Welfare Atami Hospital, Yokohama Sakae Kyosai Hospital, and Namiki Koiso Clinic) for unexplained abdominal symptoms between February 6, 2021, and May 31, 2022. The study was conducted in accordance with the principles of the Declaration of Helsinki and approved by the local ethics committee of Yokohama City University Hospital (approval number: B200800055, October 15, 2020). It was also registered with the University Hospital Medical Information Network (UMIN000043262) on February 5, 2021. All participants provided written informed consent before enrollment. Because this is a cross-sectional study aimed at exploratory investigation of the relationship between BAs in SUDD and healthy subjects, the planned enrollment was set at the number of subjects that could be enrolled within the enrollment period.

Patients with chronic abdominal symptoms were considered to have unexplained abdominal symptoms upon visiting the gastroenterology clinic at least three times during a 6-month period and did not obtain a clear diagnosis despite having undergone various tests. The patients were examined in outpatient clinics specializing in the diagnosis of chronic abdominal symptoms to rule out organic diseases such as inflammatory bowel disease or colorectal cancer. All patients underwent colonoscopy, and the presence and site of diverticulosis were assessed. Colonoscopy findings were then double-checked by authors who are expert colonoscopists. Questionnaires were used to investigate age, sex, body mass index (BMI), waist circumference, abdominal surgery history, and pain level. Abdominal pain severity was assessed using an 11-point numerical rating scale (NRS), with 0 representing no pain and 10 representing the most severe pain (17). In order to assess the shape of stool, the Bristol Stool Form Scale (BSFS) was used to grade stool consistency. Types 1 and 2 suggest constipation, types 3 and 4 indicate normal defecation, and types 5, 6, and 7 indicate diarrhea (18). Blood and fecal samples were collected, with medications pertaining to BA metabolism withdrawn for at least 1 month before sample collection.

### 2.2 Inclusion and exclusion criteria

With reference to previous reports (1), SUDD was diagnosed as follows: (1) persistence of symptoms for more than 24 h; (2) pain in the abdomen consistent with the site of the diverticulum (3) absence of signs and symptoms and clinical and microscopic evidence of acute diverticulitis; and (4) no criteria for IBS (1, 16). In Europe, left-sided diverticula are predominant, whereas right-sided diverticula are more common in Asia (16, 19). The definition of SUDD in the previous report (1) is limited to left-sided lower abdominal pain, but since the existence of right-sided SUDD has been reported in Asia (2), we modified the definition of SUDD in this report to include abdominal pain consistent with the site of the diverticulum, so that right-sided SUDD can also be diagnosed. CT or lower gastrointestinal endoscopy confirmed the absence of obvious intestinal inflammatory findings. IBS was defined according to the Rome IV criteria as recurrent abdominal pain, averaging at least 1 day per week over the last 3 months, meeting two or more of the following criteria: (1) abdominal pain associated with defecation, (2) abdominal pain associated with changes in defecation frequency, and (3) abdominal pain associated with changes in fecal shape (20). Conversely, patients with SUDD are characterized by abdominal pain that is not associated with defecation and persists for long durations, such that they do not satisfy the Rome IV criteria (1). Probiotics, antidiarrheals, antimicrobials, and ursodeoxycholic acid and cholestimide, which may affect BA kinetics, were discontinued at least 4 weeks before specimen collection. Laxatives continued to be taken internally, but patients taking elobixibat were excluded from the study because elobixibat affects BA kinetics.

Patients who had diverticula but did not fulfill the criteria for SUDD were referred to as non-SUDD patients. Patients with post-diverticulitis SUDD (PD-SUDD) are those who have experienced at least one episode of acute diverticulitis with or without complications and have abdominal pain despite resolution of macroscopic findings of diverticulitis (1); in this study, PD-SUDD patients were included in SUDD patients. SUDD patients with no history of diverticulitis were described as non-PD-SUDD. The healthy control (HC) group included individuals with no history of medications or evidence of colonic diverticula. In total, 34 patients met the eligibility criteria for the present study and had available BA measurements (UMIN000020917, approval number: B160101015, February 5, 2016).

### 2.3 Clinical and laboratory evaluations

Information pertaining to this is described in the Supplementary Methods.

### 2.4 Diverticula inflammation and complications assessment (DICA) score

Information pertaining to this is described in the Supplementary Methods.

### 2.5 BA analyses

Bile acid was measured in accordance with a previous report (21). The following is a description of the method used to measure fecal BAs. To 20 mg of fecal sample, 250 μL of 1 M potassium hydroxide solution was added, vortexed, and incubated at 80°C for 20 min. To the 5-ml tube, 2 ml of 0.5 M Potassium Phosphate Buffer (PPB) was added, and 2 μL each of 10 internal standard solutions were added. 100 μL of stool sample volume was added to the 5-mL tube containing PPB, vortexed, and placed in a Bond Elut C18 Cartridge to extract BA. The sample was evaporated to dryness in EYELA blown concentrator for 40 min, 200 μl of 0.05% acetic acid solution and acetonitrile were added, and centrifuged. 40 μL of the supernatant was quantified by liquid chromatography-mass spectrometer (Shimadzu Corporation, Kyoto, Japan). The following is a description of the method used to measure serum BAs. Ten μl of sample serum was vortexed with 2 μL each of 10 different internal standard solutions, 30 μL of 1 M hydrochloric acid, and 1000 μL of acetonitrile, and then centrifuged. The 1000 μL supernatant was evaporated to dryness for 1 h, and 200 μL of methanol was added and centrifuged. Fifty microliters of the supernatant was quantified by liquid chromatography-mass spectrometer (Shimadzu Corporation, Kyoto, Japan).

### 2.6 Statistical analyses

Data were analyzed using JMP software (version 15.0; SAS Institute Inc., Cary, NC, USA) and are expressed as mean ± standard deviation unless otherwise indicated. Student’s *t*-tests were used to compare continuous variables or univariate means between two groups; for the *F*-test, *P*-values were calculated using one-way analysis of variance across the three groups. According to Tukey’s honest significant difference *post-hoc* test to correct for multiple testing, two-sample *t*-tests were conducted for each pairwise comparison among the three groups if the *P*-value for the *F*-test was significant at a two-sided significance level of 5%. The χ^2^ test was used to compare binary variables. All *t*-tests, *F*-tests, and χ^2^ tests were two-sided with a significance level of 5% (*P* < 0.05). The graphs were prepared using GraphPad Prism 9 (GraphPad Software Inc., San Diego, CA, USA); data are presented as means ± standard errors.

## 3 Results

### 3.1 Study participants

**FIGURE 1:**
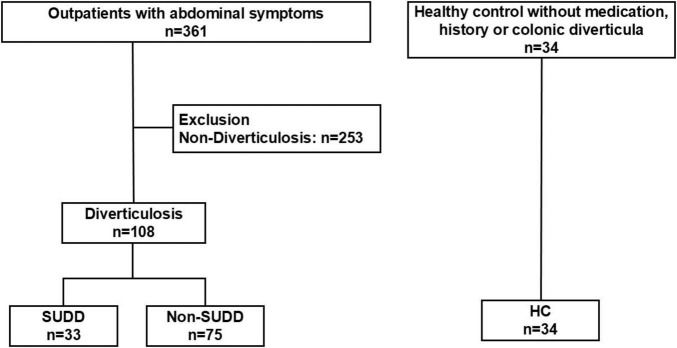
Study flow and prevalence of SUDD in patients with diverticulosis. SUDD, symptomatic uncomplicated diverticular disease; CIPO, chronic intestinal pseudo-obstruction; IBS, irritable bowel syndrome; HC, healthy controls.

In total, 361 participants with abdominal complaints were screened. In 253 individuals, including 54 with chronic intestinal pseudo-obstruction, 128 with functional constipation, 12 with functional diarrhea, and 25 with IBS, diverticulosis was ruled out. Of the 361 patients, 108 (30%) had diverticulosis, and 33 (9%) presented with SUDD. Of the 108 patients with diverticulosis, 33 (31%) had SUDD, while 75 (69%) had non-SUDD. The non-SUDD group included patients with diverticula but not meeting the diagnostic criteria for SUDD. Of the 75 patients with non-SUDD, 39 had asymptomatic diverticulosis. There were 26 patients who did not meet the criteria for SUDD but had abdominal pain symptoms, 5 had functional constipation, 2 had IBS, and 19 were other. The HC included 34 samples for which BAs were measurable from previous study samples (Figure 1).

### 3.2 Demographic and SUDD characteristics

Patients with SUDD had a mean age of 62 years, BMI of 23.6 kg/m^2^, and waist circumference of 83.4 cm. There were no differences in age, sex, BMI, or waist circumference among HC, non-SUDD, and SUDD groups. No patient had a history of ileal terminal surgery. No one in the SUDD group or the healthy group had ever had cholelithiasis or cholecystectomy. Among the patients in the non-SUDD group, two patients had cholelithiasis and two patients had a history of cholecystectomy surgery. Of the 33 patients with SUDD, 39% (13 patients) were right-sided, 45% (15 patients) were left-sided, and 15% (5 patients) were bilateral. The location of the diverticulum did not differ significantly between non-SUDD and SUDD groups. NRS scores were significantly higher in the SUDD group than in the non-SUDD group (non-SUDD 3.6 vs. SUDD 5.8, *P* < 0.0001). BSFS scores were significantly higher in the SUDD group than in the non-SUDD group (non-SUDD 3.8 vs. SUDD 5.5, *P* < 0.0001). The number of patients with a BSFS score of 6 or 7 was also significantly higher in the SUDD group than in the non-SUDD group (non-SUDD 12% vs. SUDD 63%, *P* < 0.0001). The total DICA classification score was significantly higher in the SUDD group than in the non-SUDD group (non-SUDD 1.6 vs. SUDD 2.4, *P* < 0.0001) (Table 1).

**TABLE 1:** Differences of characteristics in the HC, non-SUDD, and SUDD groups.

Variables	HC (H)	Non-SUDD (N)	SUDD (S)	*P*-value
	(*n* = 34)	(*n* = 75)	(*n* = 33)	for *F*-test or *t*-test
Age	62 (13)	65 (11)	62 (13)	0.240
Male	16 (57)	48 (68)	16 (52)	0.088
BMI (kg/m2)	23.4 (0.80)	25.0 (4.6)	23.6 (3.9)	0.090
Waist circumference (cm)	83.0 (1.9)	87.5 (12.9)	83.4 (10.4)	0.084
**Site of diverticula**				
Right side, *n* (%)	–	37 (49)	13 (39)	0.340
Left side, *n* (%)	–	20 (27)	15 (45)	0.055
Bilateral side, *n* (%)	–	18 (24)	5 (15)	0.301
Severity of abdominal pain (NRS)	–	3.6 ± 2.0	5.8 ± 2.4	<0.0001
**Stool form**				
BSFS	–	3.8 ± 1.4	5.5 ± 1.1	<0.0001
Proportion of BSFS 6 + 7, *n* (%)	–	9 (12)	21 (64)	<0.0001
**DICA classification**				
Diverticular location	–	1.5 ± 0.5	1.6 ± 0.5	0.345
Number of diverticula	–	0.1 ± 0.3	0.8 ± 0.4	<0.0001
Inflammations	–	0	0	
Complications	–	0	0	
DICA total point	–	1.6 ± 0.6	2.4 ± 0.6	<0.0001
DICA1: 1–3 points	–	75 (100)	33 (100)	
DICA 2: 4–7 points	–	0	0	
DICA 3: > 7 points	–	0	0	

Data are shown in mean (standard deviation) or number (%). BMI, body mass index; BSFS, Bristol stool form scale; NRS, numerical rating scale; DICA, Diverticular Inflammation and Complication Assessment; HC, healthy control; SUDD, symptomatic uncomplicated diverticular disease.

### 3.3 Analysis of fecal BA in the HC, non-SUDD, and SUDD groups

**FIGURE 2:**
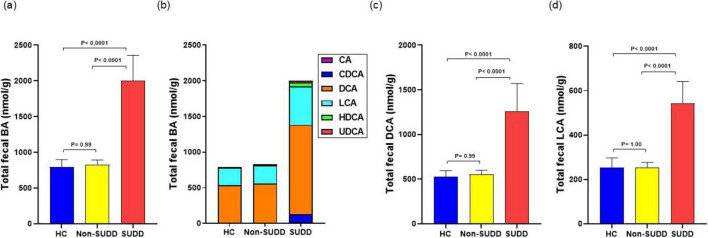
Comparison of total fecal BA levels in HC, non-SUDD, and SUDD groups. **(a)** Fecal BA levels. **(b)** Fraction types of BAs. **(c)** Fecal DCA levels. **(d)** Fecal LCA levels. BA, bile acid; CA, cholic acid; CDCA, chenodeoxycholic acid; DCA, deoxycholic acid; LCA, lithocholic acid; HDCA, hyodeoxycholic acid; UDCA, ursodeoxycholic acid; HC, healthy controls; SUDD, symptomatic uncomplicated diverticular disease.

**FIGURE 3:**
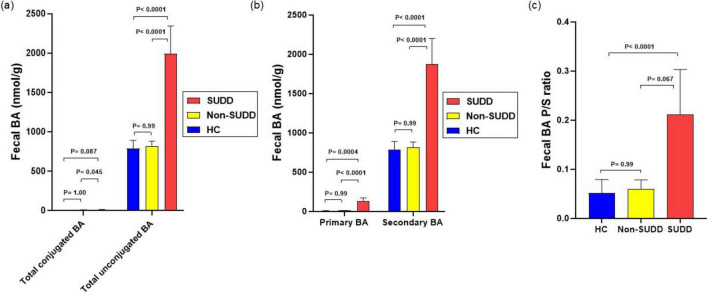
Detailed comparison of fecal BA levels in HC, non-SUDD, and SUDD groups. **(a)** Total conjugated and unconjugated BAs. **(b)** Primary and secondary BAs. **(c)** Fecal primary/secondary BA ratio. BA, bile acid; HC, healthy controls; SUDD, symptomatic uncomplicated diverticular disease.

The total BA concentration did not differ between the HC and non-SUDD groups; however, it was significantly higher in the SUDD group than in the other two groups (Figure 2a). Most fecal BAs were secondary BAs such as deoxycholic acid (DCA) and lithocholic acid (LCA) (Figure 2b). Total cholic acid (CA), chenodeoxycholic acid (CDCA), DCA, LCA, hyodeoxycholic acid (HDCA), and ursodeoxycholic acid (UDCA) levels were not different between the HC and non-SUDD groups; however, they were significantly higher in the SUDD group than in the HC and non-SUDD groups (Figures 2c, d). The same results were obtained for total unconjugated, primary, and secondary BA levels (Figures 3a, b) and the primary/secondary BA ratio (Figure 3c and Supplementary Table 1). A sensitivity analysis was performed after excluding two patients with cholelithiasis and two patients who had undergone cholecystectomy, and the results were similar to those of the main analysis. In a comparison of asymptomatic diverticula and non-SUDD with abdominal pain and SUDD, the concentrations of fecal total BA, unconjugated BA, primary BA, secondary BA, Total CA, CDCA, DCA, LCA, HDCA, and UDCA were significantly higher in the SUDD group than in the other two groups. There was no significant difference between the asymptomatic diverticula group and the non-SUDD group with abdominal pain (Supplementary Table 2). In a comparison of non-diverticula with abdominal pain, non-SUDD with abdominal pain, and SUDD, there was no significant difference in total BA concentration between the non-diverticula with abdominal pain group and the non-SUDD with abdominal pain group, but it was significantly higher in the SUDD group than in the other two groups. Total CA, total CDCA, total DCA, total LCA, Total UDCA concentrations were significantly higher in the SUDD group than in the non-diverticula with abdominal pain group (Supplementary Table 3).

### 3.4 Detailed analysis of fecal BA in the HC, non-SUDD, and SUDD groups

The levels of unconjugated CA, CDCA, DCA, LCA, HDCA, and UDCA were higher in the SUDD group than in the HC and non-SUDD groups, with no difference between the HC and non-SUDD groups. Conjugated levels of CA and UDCA did not differ between the HC and non-SUDD groups; however, they were significantly increased in the SUDD group. Conversely, the levels of conjugated DCA, LCA, and HDCA did not differ across the three groups (Supplementary Table 4).

### 3.5 Analysis of fecal BA between non-PD-SUDD and PD-SUDD groups

Among the 33 SUDD patients, 23 had non-PD-SUDD and 10 had PD-SUDD. Fecal BA concentrations were analyzed between the non-PD-SUDD and PD-SUDD groups. Total fecal BA, CA, CDCA, DCA, LCA, HDCA, and UDCA levels were not different between the non-PD-SUDD and PD-SUDD groups. Total conjugated, unconjugated, primary, secondary BA levels and the primary/secondary BA ratio did not differ between the non-PD-SUDD and PD-SUDD groups (Supplementary Table 5).

### 3.6 Analysis of serum BA in the HC, non-SUDD, and SUDD groups

Total serum BA levels did not differ across the three groups (Supplementary Figure 1). Most serum BAs were UDCA (Supplementary Figure 2a). CA, CDCA, DCA, LCA, HDCA, and UDCA levels were not different between the non-SUDD and SUDD groups (Supplementary Table 6). Total conjugated, unconjugated, primary, and secondary BA levels did not differ between the non-SUDD and SUDD groups (Supplementary Figures 2b, c). Conjugated and unconjugated levels of CA, CDCA, DCA, LCA, HDCA, and UDCA did not differ between the non-SUDD and SUDD groups (Supplementary Table 7). The serum primary/secondary BA ratio did not differ between the HC and non-SUDD groups; however, it was significantly lower in the SUDD group than in the HC group (Supplementary Figure 2d).

### 3.7 Analysis of BAs according to diverticular site in the SUDD group

No differences in fecal BA concentrations were found among patients with right-sided SUDD, left-sided SUDD, and bilateral SUDD (Supplementary Table 8). Serum BA concentrations also did not differ according to site (Supplementary Table 9).

### 3.8 Comparison of blood and fecal test findings

**FIGURE 4:**
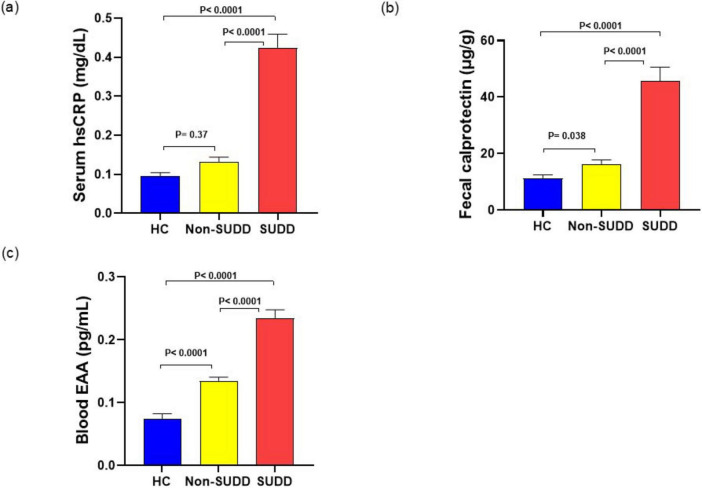
Inflammatory markers in HC, non-SUDD, and SUDD groups. **(a)** Serum hsCRP. **(b)** Fecal calprotectin. **(c)** Blood EAA. EAA, Endotoxin activity assay; HC, healthy controls; hsCRP, High-sensitivity C-reactive protein; SUDD, symptomatic uncomplicated diverticular disease.

**FIGURE 5:**
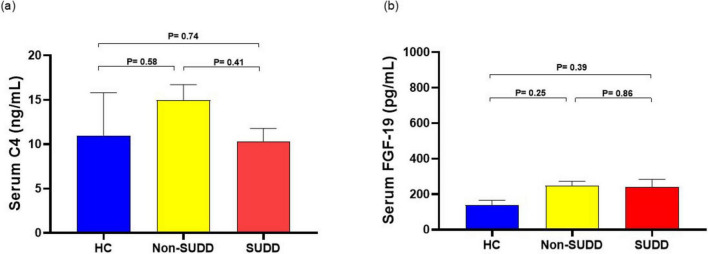
Comparison of serum C4 and FGF-19 levels in HC, non-SUDD, and SUDD groups. **(a)** Serum C4. **(b)** Serum FGF-19. FGF-19, fibroblast growth factor 19; HC, healthy controls; SUDD, symptomatic uncomplicated diverticular disease.

Serum high-sensitivity C-reactive protein (hsCRP) levels were significantly higher in the SUDD group than in the HC and non-SUDD groups; however, they did not differ between the non-SUDD and HC groups (Figure 4a and Supplementary Table 10). Fecal calprotectin levels were significantly higher in the SUDD and non-SUDD groups than in the HC group, and in the SUDD group than in the non-SUDD group (Figure 4b). Blood Endotoxin Activity Assay (EAA) levels were significantly higher in the SUDD group than in the other groups, and higher in the non-SUDD group than in the HC group (Figure 4c). Serum C4 and FGF-19 levels did not differ across the three groups (Figure 5).

### 3.9 Correlation between inflammatory markers and fecal BAs

The fecal total BA concentration was mildly positively correlated with fecal calprotectin and blood EAA (vs fecal calprotectin; *r* = 0.28, vs blood EAA; *r* = 0.23). Inflammatory markers were more positively correlated with secondary BAs than with primary BAs and more positively correlated with unconjugated BAs than with conjugated BAs (Supplementary Figures 3a, b, c, d and Supplementary Table 11). Among the unconjugated secondary BAs, UDCA most strongly correlated with inflammatory markers (Supplementary Table 12).

### 3.10 Correlation of NRS, BSFS and DICA classification with fecal BAs

Fecal total BA concentration showed a mild positive correlation with BSFS score and a very weak positive correlation with NRS score and DICA total score. Primary BA concentrations showed a mild positive correlation with NRS score, and primary, secondary, and unconjugated BA concentrations showed a mild positive correlation with BSFS score. Fecal CA, HDCA and UDCA were mildly positively correlated with NRS score; and fecal CDCA, DCA, LCA, HDCA and UDCA were mildly positively correlated with BSFS score; and Fecal CA, LCA and UDCA were mildly positively correlated with DICA total score (Supplementary Table 13).

## 4 Discussion

While increased levels of fecal BAs, especially secondary BAs, have been reported to induce intestinal inflammation and diarrhea (11, 12), this was the first study to investigate the association between SUDD and fecal BAs. Our results showed that fecal BA levels in patients with SUDD were higher than those in the HC and non-SUDD groups, especially with regard to unconjugated BAs. Total BA concentration was significantly higher in the SUDD group than in the group of non-diverticula with abdominal pain and non-SUDD with abdominal pain. Since fecal BA concentrations do not increase in patients with abdominal pain without diverticula or in patients with abdominal pain and diverticula but who do not meet the diagnostic criteria for SUDD, the elevation of fecal BA concentrations suggests that it is characteristic of SUDD among patients with abdominal pain. Additionally, we observed higher levels of fecal calprotectin and serum hsCRP in patients with SUDD than in the non-SUDD group. Our results suggest that the symptoms of abdominal pain and diarrhea in patients with SUDD may be caused by inflammation-inducing effects of BAs in the gut. Secondary BAs are particularly cytotoxic and may strongly influence abdominal symptoms in patients with SUDD (22).

Herein, blood EAA levels were higher in both diverticulum groups than in the HC group. Two possible intestinal factors cause high blood endotoxin levels; these include quantitative abnormalities (abnormal endotoxin-producing bacteria) and increased influx (increased influx due to intestinal barrier failure), and intestinal permeability is increased in SUDD (23–25). BAs have increasing hydrophobicity (UDCA < CA < CDCA < DCA < LCA) and relate to hydrophobicity and toxicity. LCA, thought to be the most hydrophobic BA, can affect intestinal permeability and boost its blood diffusion (26, 27). BAs alter the intestinal microbiota and disrupt the intestinal barrier (28, 29). Thus, the increase in blood endotoxins in patients with SUDD may be due to increased intestinal permeability caused by changes in the intestinal microbiota and disruption of the intestinal barrier because of increased fecal BAs in patients with SUDD. Therefore, it is important to measure the gut microbiota and intestinal barrier function in patients with and without SUDD. In the present study, fecal unconjugated UDCA showed the strongest positive correlation with fecal calprotectin and blood EAA, although the proportion was small among the fecal BAs. Because UDCA has an attenuative effect on colon inflammation (30), it was suggested that fecal UDCA is increased in patients with high levels of colon inflammation.

We also focused on the relationship between pain and endotoxin levels, since endotoxins decrease the pain threshold (31) and found that pain was more intense in the SUDD group than in the non-SUDD group. Thus, higher endotoxin levels may have reduced the pain threshold and made patients feel more pain in SUDD groups.

Although fecal BA levels were higher in the SUDD group than in the HC and non-SUDD groups, serum C4 and FGF-19 levels in the SUDD group did not differ significantly among the three groups. This suggests that the bile synthesis capacity did not increase in patients with SUDD, and that these participants may have had less BA absorption from the terminal ileum. The relationship between diverticular disease and small intestinal bacterial overgrowth has been shown in a report by Tursi et al. (32). Thus, an increase in BAs in the colonic tract may influence gut bacterial overgrowth. A decrease in the serum primary/secondary BA ratio was observed in patients with SUDD. In patients with SUDD, primary BA reabsorption in the ileal BA transporter is decreased, resulting in lower serum primary BA concentrations, and diffusion is increased in the colon, which is thought to increase serum secondary BA concentrations. The micro inflammatory effects of raised colonic BAs cause diarrhea, elevated fecal calprotectin, and elevated blood endotoxin levels due to increased intestinal permeability.

**FIGURE 6:**
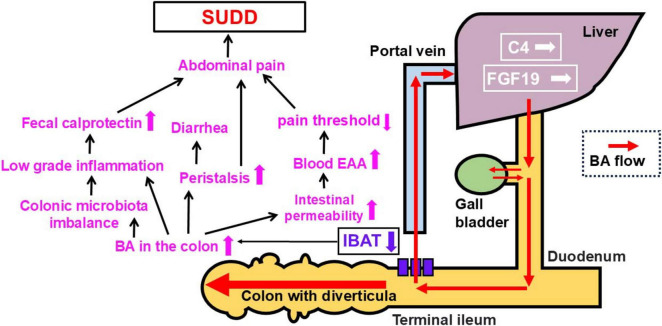
Patho-physiology hypothesis on SUDD (see the text for further explanations). BA, bile acid; IBAT, ileal bile acid transporter; C4, 7α-hydroxy-4-cholesten-3-one; EAA, Endotoxin activity assay; FGF-19, fibroblast growth factor 19; SUDD, symptomatic uncomplicated diverticular disease.

We did not find enhanced production of BAs in SUDD. However, thinking about the pathogenesis of SUDD symptoms, we can hypothesized the following role of the BAs (see Figure 6): (1) raised BAs colonic concentrations may cause significant changes in colonic microbiota with intestinal microinflammation, confirmed by raised fecal calprotectin; (2) they can also increase the intestinal permeability, which causes elevated blood endotoxin levels and lower pain threshold; (3) BAs may increase the peristalsis (31); The role of low grade inflammation and the gut microbiota imbalance associated to BAs is strengthened by the recent demonstration that acute diverticulitis has shown a correlation between BAs (primary BAs such as CA and CDCA and the secondary BA HydroDCA) and some gut microbiota taxa (*Firmicutes bacterium* CAG 41, *Anaerostipes hadus*, *Bilophila wadsworthia*, *Parabacteroidetes sistasonis*) (33). The pathogen *Bilophila wadsworthia* proliferates in response to Western diets, causing intestinal inflammation and abnormal BA metabolism (34, 35). In controls, *Bilophila wadsworthia* was positively correlated with alpha-muricholic acid and cholic acid, but inversely correlated in diverticulitis. It is argued that the interplay between *Bilophila wadsworthia* and BAs may influence the pathogenesis of diverticulitis (33). Since significant microbiota imbalance has been detected also in SUDD, with abundance of pathobionts (36), the future challenge is to clarify the gut microbiota profile according to high fecal BA in SUDD. All these findings suggest that BAs - mainly secondary BAs (DCA and LCA) - may be part of the complex mechanisms inducing intestinal inflammation in SUDD. This hypothesis is confirmed by the detection of a significant correlation between BAs levels and raised calprotectin levels in SUDD. The strengths of this study are as follows. First, this was the first study to examine the relationship between fecal BAs and SUDD. Second, it was a multicenter study. Third, it was conducted by analyzing different subtypes of BAs. The limitations are as follows. First, the sample size was small. Second, only Japanese patients were evaluated. Third, the participants were restricted to outpatients and their families. Fourth, intestinal bacteria were not measured. Fifth, the association between SUDD and BAs is clear; however, it is unclear which is the cause and which is the consequence. In the future, it would be desirable to elucidate the relationship between fecal BAs and SUDD in endoscopically screened patients and to conduct an analytical study of fecal BAs in patients with SUDD, including those in Europe. In addition, the exact mechanisms underlying the development of SUDD and increase in fecal BA concentrations need to be clarified in clinical and basic research, also exploring whether reducing fecal BA levels may be beneficial for patients with SUDD.

## Data Availability

The raw data supporting the conclusions of this article will be made available by the authors, without undue reservation.
