# Supplementary material for: Patients with symptomatic uncomplicated diverticular disease have high fecal bile acid concentrations
Source: Front Med (Lausanne). 2025 Jul 9;12:1533644. doi: 10.3389/fmed.2025.1533644 (PMC12283573; doi:10.3389/fmed.2025.1533644)
Supplement: Supplementary file 1 [file Data_Sheet_1.docx]

Supplementary Material

# Supplementary Methods

## Clinical and laboratory evaluation

Blood endotoxin activity assays (EAAs) were carried out as previously described (1-3). Venous blood samples were obtained after the patients had fasted overnight (12 h) for measurement of high-sensitivity C-reactive protein (hsCRP) levels at each institution. The normal range for hsCRP is 0.3 mg/dL. Fecal and blood specimens were collected at each facility and promptly stored at −80°C. The stored specimens were transported to Yokohama City University Hospital, where fecal calprotectin, serum 7α-hydroxy-4-cholesten-3-one (C4), and serum fibroblast growth factor 19 (FGF-19) measurements were performed at a single institution. Fecal calprotectin levels were measured using an enzyme-linked immunosorbent assay (ELISA) kit, according to the manufacturer’s instructions (HK382, Hycult Biotech Inc., Wayne, PA, USA). C4 levels were measured as previously described (4), and serum FGF-19 levels were assayed using an ELISA kit (RD191107200R, BioVendor R&D Inc., Brno, Czech Republic).

## Diverticula Inflammation and Complications Assessment (DICA) score

Developed and validated in 2015, the DICA score represents the first endoscopic classification of diverticulosis (5). The DICA enables evaluation of the severity of diverticulosis, whether left or right, by considering several factors including the extension of diverticulosis, number of diverticula in each region, presence of inflammatory signs, and occurrence of complications (6). Diverticulosis of the left colon scored 2 points, while diverticulosis of the right colon scored 1 point. Each region's diverticula count was ranked as follows: up to 15 (grade I: 0 points) and >15 (grade II: 1 point). Inflammation was evaluated by assigning 1 point to edema/hyperemia, 2 points to erosions, and 3 points to segmental colitis. Complications, including rigidity of the colon, the presence of purulent material exiting the diverticular opening, and bleeding, were assessed. DICA scores were divided into three categories: DICA 1 (up to 3 points), reflecting simple diverticulosis; DICA 2 (4 to 7 points), reflecting conditions ranging from severe diverticulosis to milder diverticulitis; and DICA 3 (more than 7 points), reflecting more severe diverticulitis or complications following diverticulitis (i.e., stenosis) (7).

# Supplementary Figures and Tables

## Supplementary Figures

**Supplementary Figure 1.** Comparison of total serum BA levels among HC, non-SUDD, and SUDD groups. BA, bile acid; SUDD, symptomatic uncomplicated diverticular disease

**(a)**

**(b)**

**(c)**

**(d)**

**Supplementary Figure 2. Detailed comparison of serum BA levels among HC, non-SUDD, and SUDD groups.** BA, bile acid; CA, cholic acid; CDCA, chenodeoxycholic acid; conj, conjugated; DCA, deoxycholic acid; HC, healthy controls; HDCA, hyodeoxycholic acid; LCA, lithocholic acid; P/S, primary bile acid/secondary bile acid; SUDD, symptomatic uncomplicated diverticular disease; UDCA, ursodeoxycholic acid; unconj, unconjugated

**(a)　　　　　　　　　　　　　　　　　　　　(b)**


**(c) 　 　 (d)**

**Supplementary Figure 3. Correlation of inflammatory markers with fecal BAs.** BA, bile acid; conj, conjugated; EAA, endotoxin activity assay; unconj, unconjugated

## Supplementary Tables

**Supplementary Table 1. Fecal BA concentration in the HC, non-SUDD, and SUDD groups**

| Bile Acid | | HC (H) | Non-SUDD (N) | SUDD (S) | P-value | P-value | | |
| --- | --- | --- | --- | --- | --- | --- | --- | --- |
| (μmol/g) | | (n=34) | (n=75) | (n=33) | for F test | H vs N | H vs S | N vs S |
| *Total BA* | | 795 (599) | 827 (564) | 2003 (2044) | <0.0001 | 0.99 | <0.0001 | <0.0001 |
|  | Total CA | 3 (6) | 3 (7) | 20 (25) | <0.0001 | 0.98 | <0.0001 | <0.0001 |
|  | Total CDCA | 4 (6) | 7 (13) | 107 (249) | 0.0002 | 1.00 | 0.002 | 0.0003 |
|  | Total DCA | 525 (411) | 551 (416) | 1256 (1813) | 0.001 | 0.99 | 0.005 | 0.001 |
|  | Total LCA | 253 (257) | 253 (209) | 541 (573) | 0.0002 | 1.00 | 0.002 | 0.0002 |
|  | Total HDCA | 8 (14) | 10 (25) | 55 (66) | <0.0001 | 0.96 | <0.0001 | <0.0001 |
|  | Total UDCA | 1 (2) | 2 (5) | 23 (26) | <0.0001 | 0.94 | <0.0001 | <0.0001 |
|  |  |  |  |  |  |  |  |  |
|  | Total conj BA | 5 (4) | 5 (4) | 11 (22) | 0.040 | 1.00 | 0.087 | 0.045 |
|  | Total unconj BA | 790 (597) | 822 (564) | 1992 (2025) | <0.0001 | 0.99 | <0.0001 | <0.0001 |
|  |  |  |  |  |  |  |  |  |
|  | Primary BA | 7 (11) | 10 (19) | 127 (256) | <0.0001 | 0.99 | 0.0004 | <0.0001 |
|  | Secondary BA | 788 (601) | 817 (570) | 1876 (1859) | <0.0001 | 0.99 | <0.0001 | <0.0001 |
|  | P/S ratio | 0.05 (0.2) | 0.06 (0.2) | 0.2 (0.5) | 0.031 | 0.99 | 0.067 | 0.037 |

Data are shown as mean (standard deviation).

BA, bile acid; CA, cholic acid; CDCA, chenodeoxycholic acid; conj, conjugated; DCA, deoxycholic acid; HC, healthy controls; HDCA, hyodeoxycholic acid; LCA, lithocholic acid; P/S, primary bile acid/secondary bile acid; SUDD, symptomatic uncomplicated diverticular disease; UDCA, ursodeoxycholic acid; unconj, unconjugated

**Supplementary Table 2. Fecal BA concentrations in the asymptomatic diverticula, non-SUDD with abdominal pain, and SUDD groups**

| Bile Acid | | Asymptomatic diverticula (A) | Non-SUDD with abdominal pain (O) | SUDD (S) |  | p-value | p-value | | |
| --- | --- | --- | --- | --- | --- | --- | --- | --- | --- |
| (μmol/g) | | (n=45) | (n=30) | (n=33) |  | for F test | A vs O | A vs S | O vs S |
| *Total BA* | | 900 (603) | 716 (491) | 2003 (2044) |  | < 0.0001 | 0.80 | 0.0004 | 0.0002 |
|  | Total CA | 1 (2) | 6 (11) | 20 (25) |  | < 0.0001 | 0.36 | < 0.0001 | 0.002 |
|  | Total CDCA | 4 (5) | 10 (19) | 107 (249) |  | 0.0032 | 0.98 | 0.004 | 0.018 |
|  | Total DCA | **612 (436)** | 461 (373) | 1256 (1813) |  | 0.007 | 0.82 | 0.025 | 0.010 |
|  | Total LCA | 275 (211) | 220 (204) | 541 (573) |  | 0.0009 | 0.79 | 0.005 | 0.0017 |
|  | Total HDCA | 6 (9) | 17 (37) | 55 (66) |  | < 0.0001 | 0.50 | < 0.0001 | 0.0012 |
|  | Total UDCA | 2 (6) | 3 (3) | 23 (26) |  | < 0.0001 | 0.99 | < 0.0001 | < 0.0001 |
|  |  |  |  |  |  |  |  |  |  |
|  | Total conj BA | 5 (4) | 6 (4) | 11 (22) |  | 0.105 |  |  |  |
|  | Total unconj BA | 895 (602) | 711 (489) | 1992 (2025) |  | < 0.0001 | 0.80 | 0.0004 | 0.0002 |
|  |  |  |  |  |  |  |  |  |  |
|  | Primary BA | 6 (6) | 17 (28) | 127 (256) |  | 0.0007 | 0.99 | 0.0004 | < 0.0001 |
|  | Secondary BA | 894 (605) | 700 (499) | 1876 (1859) |  | < 0.0001 | 0.75 | 0.0007 | 0.0002 |
|  | P/S ratio | 0.02 (0.08) | 0.1 (0.2) | 0.2 (0.5) |  | 0.039 | 0.42 | 0.030 | 0.48 |

Data are shown as mean (standard deviation).

BA, bile acid; CA, cholic acid; CDCA, chenodeoxycholic acid; conj, conjugated; DCA, deoxycholic acid; HC, healthy controls; HDCA, hyodeoxycholic acid; LCA, lithocholic acid; P/S, primary bile acid/secondary bile acid; SUDD, symptomatic uncomplicated diverticular disease; UDCA, ursodeoxycholic acid; unconj, unconjugated

**Supplementary Table 3. Fecal BA concentrations in non-diverticula with abdominal pain, non-SUDD with abdominal pain and SUDD groups**

| Bile Acid | | Non-diverticula with abdominal pain (ND) | non-SUDD with abdominal pain (O) | SUDD (S) |  | p-value | p-value | | |
| --- | --- | --- | --- | --- | --- | --- | --- | --- | --- |
| (μmol/g) | | (n=86) | (n=30) | (n=33) |  | for F test | ND vs O | ND vs S | O vs S |
| *Total BA* | | 908 (686) | 716 (491) | 2003 (2044) |  | < 0.0001 | 0.70 | < 0.0001 | < 0.0001 |
|  | Total CA | 7 (12) | 6 (11) | 20 (25) |  | 0.0002 | 0.99 | 0.0002 | 0.003 |
|  | Total CDCA | 37 (59) | 10 (19) | 107 (249) |  | 0.006 | 0.59 | 0.02 | 0.008 |
|  | Total DCA | 816 (667) | 461 (373) | 1256 (1813) |  | 0.008 | 0.22 | 0.09 | 0.006 |
|  | Total LCA | 42 (42) | 220 (204) | 541 (573) |  | < 0.0001 | 0.01 | < 0.0001 | < 0.0001 |
|  | Total HDCA | 14 (24) | 17 (37) | 55 (66) |  | < 0.0001 | 0.93 | < 0.0001 | 0.0005 |
|  | Total UDCA | 3 (5) | 3 (3) | 23 (26) |  | < 0.0001 | 1.00 | < 0.0001 | < 0.0001 |

Data are shown as mean (standard deviation).

BA, bile acid; CA, cholic acid; CDCA, chenodeoxycholic acid; conj, conjugated; DCA, deoxycholic acid; HDCA, hyodeoxycholic acid; LCA, lithocholic acid; SUDD, symptomatic uncomplicated diverticular disease; UDCA, ursodeoxycholic acid.

**Supplementary Table 4. Fecal BA concentrations in the HC, non-SUDD, and SUDD groups divided into detailed categories**

| Bile Acid | | HC (H) | Non-SUDD (N) | SUDD (S) | P-value | P-value | | |
| --- | --- | --- | --- | --- | --- | --- | --- | --- |
| (μmol/g) | | (n=84) | (n=75) | (n=33) | for F-test | H vs N | H vs S | N vs S |
| Total CA | |  |  |  |  |  |  |  |
|  | Unconj CA | 2 (6) | 3 (7) | 18 (24) | <0.0001 | 0.99 | <0.0001 | <0.0001 |
|  | Conj CA | 0.6 (1) | 0.7 (1) | 2 (3) | 0.008 | 0.94 | 0.018 | 0.012 |
| Total CDCA | |  |  |  |  |  |  |  |
|  | Unconj CDCA | 4 (6) | 6 (12) | 106 (247) | 0.0002 | 1.00 | 0.002 | 0.0003 |
|  | Conj CDCA | 0.2 (0.1) | 0.3 (0.7) | 0.9 (2) | 0.019 | 0.90 | 0.032 | 0.031 |
| Total DCA | |  |  |  |  |  |  |  |
|  | Unconj DCA | 521 (410) | 548 (415) | 1249 (1794) | 0.001 | 0.99 | 0.005 | 0.001 |
|  | Conj DCA | 4 (3) | 3 (3) | 7 (19) | 0.14 |  |  |  |
| Total LCA | |  |  |  |  |  |  |  |
|  | Unconj LCA | 253 (257) | 253 (209) | 541 (573) | 0.0002 | 1.00 | 0.002 | 0.0002 |
|  | Conj LCA | 0.03 (0.04) | 0.03 (0.03) | 0.04 (0.03) | 0.25 |  |  |  |
| Total HDCA | |  |  |  |  |  |  |  |
|  | Unconj HDCA | 7 (14) | 10 (25) | 55 (66) | <0.0001 | 0.96 | <0.0001 | <0.0001 |
|  | Conj HDCA | 0.5 (0.3) | 0.5 (0.4) | 0.4 (0.1) | 0.15 |  |  |  |
| Total UDCA | |  |  |  |  |  |  |  |
|  | Unconj UDCA | 1 (2) | 2 (5) | 23 (25) | <0.0001 | 0.94 | <0.0001 | <0.0001 |
|  | Conj UDCA | 0.04 (0.09) | 0.06 (0.2) | 0.3 (0.5) | 0.0007 | 1.00 | 0.003 | 0.001 |

Data are shown as mean (standard deviation).

BA, bile acid; CA, cholic acid; CDCA, chenodeoxycholic acid; Conj, conjugated; DCA, deoxycholic acid; HC, healthy control; HDCA, hyodeoxycholic acid; LCA, lithocholic acid; SUDD, symptomatic uncomplicated diverticular disease; UDCA, ursodeoxycholic acid; Unconj, unconjugated

**Supplementary Table 5. Fecal BA concentrations between non-PD-SUDD, and PD-SUDD groups**

| Bile Acid | | Non-PD-SUDD | PD-SUDD |  | p-value |
| --- | --- | --- | --- | --- | --- |
| (μmol/g) | | (n=23) | (n=10) |  |  |
| *Total BA* | | 2198 (2369) | 1553 (900) |  | 0.414 |
|  | Total CA | 19 (25) | 21 (26) |  | 0.775 |
|  | Total CDCA | 116 (289) | 86 (125) |  | 0.7580 |
|  | Total DCA | 1398 (2121) | 929 (716) |  | 0.503 |
|  | Total LCA | 574 (634) | 466 (418) |  | 0.6260 |
|  | Total HDCA | 68 (73) | 25 (31) |  | 0.082 |
|  | Total UDCA | 22 (23) | 26 (33) |  | 0.699 |
|  |  |  |  |  |  |
|  | Total conj BA | 13 (26) | 5 (3) |  | 0.344 |
|  | Total unconj BA | 2185 (2346) | 1548 (900) |  | 0.415 |
|  |  |  |  |  |  |
|  | Primary BA | 135 (293) | 108 (148) |  | 0.785 |
|  | Secondary BA | 2063 (2120) | 1446 (1005) |  | 0.389 |
|  | P/S ratio | 0.09 (0.1) | 0.5 (0.9) |  | 0.051 |

Data are shown as mean (standard deviation).

BA, bile acid; CA, cholic acid; CDCA, chenodeoxycholic acid; conj, conjugated; DCA, deoxycholic acid; HC, healthy controls; HDCA, hyodeoxycholic acid; LCA, lithocholic acid; PD-SUDD, post-diverticulitis symptomatic uncomplicated diverticular disease; P/S, primary bile acid/secondary bile acid; UDCA, ursodeoxycholic acid; unconj, unconjugated

**Supplementary Table 6. Serum BA concentrations in the HC, non-SUDD, and SUDD groups**

| Bile Acid | | HC (H) | Non-SUDD (N) | SUDD (S) |  | p-value | p-value | | |
| --- | --- | --- | --- | --- | --- | --- | --- | --- | --- |
| (μM) | | (n=21) | (n=41) | (n=23) |  | for F test | H vs N | H vs S | N vs S |
| *Total BA* | | 4.4 (2.4) | 4.9 (2.6) | 5.1 (3.1) |  | 0.638 |  |  |  |
|  | Total CA | 0.31 (0.30) | 0.24 (0.11) | 0.17 (0.10) |  | 0.035 | 0.239 | 0.026 | 0.355 |
|  | Total CDCA | 1.5 (1.2) | 1.6 (1.0) | 1.2 (0.91) |  | 0.458 |  |  |  |
|  | Total DCA | 0.96 (1.1) | 0.58 (0.10) | 0.44 (0.13) |  | 0.017 | 0.062 | 0.017 | 0.651 |
|  | Total LCA | 0.38 (0.18) | 0.38 (0.18) | 0.61 (1.2) |  | 0.298 |  |  |  |
|  | Total HDCA | 0.35 (0.17) | 0.25 (0.22) | 0.24 (0.10) |  | 0.116 |  |  |  |
|  | Total UDCA | 0.79 (0.49) | 1.9 (2.1) | 2.4 (2.1) |  | 0.020 | 0.091 | 0.017 | 0.538 |
|  |  |  |  |  |  |  |  |  |  |
|  | Total conj BA | 2.3 (2.3) | 1.4 (0.66) | 1.2 (0.41) |  | 0.010 | 0.028 | 0.012 | 0.756 |
|  | Total unconj BA | 2.1 (1.4) | 3.5 (2.5) | 4.0 (3.0) |  | 0.039 | 0.098 | 0.041 | 0.760 |
|  |  |  |  |  |  |  |  |  |  |
|  | Primary BA | 1.9 (1.3) | 1.8 (1.0) | 1.4 (0.96) |  | 0.310 |  |  |  |
|  | Secondary BA | 2.5 (1.5) | 3.1 (2.1) | 3.7 (2.5) |  | 0.179 |  |  |  |
|  | P/S ratio | 0.7 (0.43) | 0.7 (0.39) | 0.5 (0.27) |  | 0.024 | 0.860 | 0.050 | 0.036 |

Data are shown as mean (standard deviation)

BA, bile acid; CA, cholic acid; CDCA, chenodeoxycholic acid; DCA, deoxycholic acid; LCA, lithocholic acid; HDCA, hyodeoxycholic acid; UDCA, ursodeoxycholic acid; Conj, conjugated; Unconj, unconjugated; P/S, primary bile acid/secondary bile acid; HC, healthy control; SUDD, symptomatic uncomplicated diverticular disease

**Supplementary Table 7. Serum BA concentrations in the HC, non-SUDD, and SUDD groups divided into detailed categories**

| Bile Acid | | HC (H) | Non-SUDD (N) | SUDD (S) |  | p-value | p-value | | |
| --- | --- | --- | --- | --- | --- | --- | --- | --- | --- |
| (μM) | | (n=21) | (n=41) | (n=23) |  | for F test | H vs N | H vs S | N vs S |
| Total CA | |  |  |  |  |  |  |  |  |
|  | Unconj CA | 0.19 (0.25) | 0.07 (0.02) | 0.03 (0.03) |  | 0.001 | 0.004 | 0.001 | 0.617 |
|  | Conj CA | 0.12 (0.07) | 0.17 (0.08) | 0.14 (0.06) |  | 0.029 | 0.025 | 0.498 | 0.327 |
| Total CDCA | |  |  |  |  |  |  |  |  |
|  | Unconj CDCA | 0.79 (0.58) | 1.1 (0.82) | 0.92 (0.83) |  | 0.305 |  |  |  |
|  | Conj CDCA | 0.76 (1.1) | 0.46 (0.39) | 0.32 (0.29) |  | 0.063 |  |  |  |
| Total DCA | |  |  |  |  |  |  |  |  |
|  | Unconj DCA | 0.29 (0.24) | 0.25 (0.14) | 0.20 (0.09) |  | 0.150 |  |  |  |
|  | Conj DCA | 0.67 (1.2) | 0.33 (0.25) | 0.25 (0.09) |  | 0.052 |  |  |  |
| Total LCA | |  |  |  |  |  |  |  |  |
|  | Unconj LCA | 0.31 (0.14) | 0.37 (0.18) | 0.61 (1.2) |  | 0.219 |  |  |  |
|  | Conj LCA | 0.13 (0.06) | 0.07 (0.006) | 0.07 (0.007) |  | < 0.0001 | < 0.0001 | < 0.0001 | 0.997 |
| Total HDCA | |  |  |  |  |  |  |  |  |
|  | Unconj HDCA | 0.13 (0.10) | 0.15 (0.22) | 0.14 (0.10) |  | 0.842 |  |  |  |
|  | Conj HDCA | 0.22 (0.15) | 0.10 (0.003) | 0.10 (0.003) |  | < 0.0001 | < 0.0001 | < 0.0001 | 1.000 |
| Total UDCA | |  |  |  |  |  |  |  |  |
|  | Unconj UDCA | 0.42 (0.43) | 1.6 (2.1) | 2.1 (2.1) |  | 0.012 | 0.056 | 0.011 | 0.553 |
|  | Conj UDCA | 0.37 (0.24) | 0.28 (0.04) | 0.30 (0.11) |  | 0.039 | 0.032 | 0.165 | 0.862 |

Data are shown in mean (standard deviation)

BA, bile acid; CA, cholic acid; CDCA, chenodeoxycholic acid; DCA, deoxycholic acid; LCA, lithocholic acid; HDCA, hyodeoxycholic acid; UDCA, ursodeoxycholic acid; Conj, conjugated; Unconj, unconjugated; HC, healthy control; SUDD, symptomatic uncomplicated diverticular disease

**Supplementary Table 8. Fecal BA concentrations according to site in the SUDD group**

| Bile Acid | | Right | Left | Bilateral |  | p-value |
| --- | --- | --- | --- | --- | --- | --- |
| (μmol/g) | | (n=13) | (n=15) | (n=5) |  | for F test |
| *Total BA* | | 2267 (3126) | 1776 (755) | 1996 (1301) |  | 0.827 |
|  | Total CA | 21 (24) | 19 (28) | 15 (19) |  | 0.901 |
|  | Total CDCA | 156 (375) | 66 (104) | 102 (153) |  | 0.654 |
|  | Total DCA | 1465 (2849) | 1210 (580) | 850 (638) |  | 0.815 |
|  | Total LCA | 529 (616) | 427 (283) | 917 (989) |  | 0.260 |
|  | Total HDCA | 66 (83) | 37 (36) | 82 (85) |  | 0.315 |
|  | Total UDCA | 30 (33) | 16 (14) | 30 (30) |  | 0.303 |
|  |  |  |  |  |  |  |
|  | Total conj BA | 16 (34) | 8 (8) | 5 (3) |  | 0.539 |
|  | Total unconj BA | 2250 (3094) | 1768 (753) | 1992 (1300) |  | 0.829 |
|  |  |  |  |  |  |  |
|  | Primary BA | 177 (374) | 86 (130) | 117 (171) |  | 0.654 |
|  | Secondary BA | 2090 (2803) | 1690 (728) | 1879 (1394) |  | 0.859 |
|  | P/S ratio | 0.3 (0.7) | 0.08 (0.1) | 0.3 (0.7) |  | 0.437 |

Data are shown as mean (standard deviation)

BA, bile acid; CA, cholic acid; CDCA, chenodeoxycholic acid; DCA, deoxycholic acid; LCA, lithocholic acid; HDCA, hyodeoxycholic acid; UDCA, ursodeoxycholic acid; Conj, conjugated; Unconj, unconjugated; P/S, primary bile acid/secondary bile acid; SUDD, symptomatic uncomplicated diverticular disease

**Supplementary Table 9. Serum BA concentrations according to site in the SUDD group**

| Bile Acid | | Right | Left | Bilateral |  | p-value |
| --- | --- | --- | --- | --- | --- | --- |
| (μmol/g) | | (n=9) | (n=11) | (n=3) |  | for F test |
| *Total BA* | | 5.7 (3.3) | 4.8 (3.0) | 4.9 (3.6) |  | 0.823 |
|  | Total CA | 0.2 (0.04) | 0.2 (0.1) | 0.2 (0.04) |  | 0.663 |
|  | Total CDCA | 1.2 (0.9) | 1.2 (0.8) | 1.6 (1.5) |  | 0.765 |
|  | Total DCA | 0.5 (0.2) | 0.4 (0.07) | 0.5 (0.3) |  | 0.343 |
|  | Total LCA | 0.4 (0.2) | 0.8 (1.7) | 0.5 (0.2) |  | 0.701 |
|  | Total HDCA | 0.2 (0.1) | 0.2 (0.07) | 0.3 (0.2) |  | 0.390 |
|  | Total UDCA | 3.2 (2.3) | 1.9 (2.0) | 1.7 (1.8) |  | 0.311 |
|  |  |  |  |  |  |  |
|  | Total conj BA | 1.2 (0.4) | 1.2 (0.4) | 1.1 (0.2) |  | 0.886 |
|  | Total unconj BA | 4.4 (3.4) | 3.6 (2.8) | 3.8 (3.5) |  | 0.838 |
|  |  |  |  |  |  |  |
|  | Primary BA | 1.3 (0.9) | 1.4 (0.9) | 1.8 (1.5) |  | 0.777 |
|  | Secondary BA | 4.3 (2.5) | 3.4 (2.7) | 3.1 (2.1) |  | 0.658 |
|  | P/S ratio | 0.3 (0.1) | 0.5 (0.3) | 0.6 (0.2) |  | 0.152 |

Data are shown as mean (standard deviation)

BA, bile acid; CA, cholic acid; CDCA, chenodeoxycholic acid; DCA, deoxycholic acid; LCA, lithocholic acid; HDCA, hyodeoxycholic acid; UDCA, ursodeoxycholic acid; Conj, conjugated; Unconj, unconjugated; P/S, primary bile acid/secondary bile acid; SUDD, symptomatic uncomplicated diverticular disease

**Supplementary Table 10. Blood and fecal tests in HC, non-SUDD, and SUDD groups**

| Variables | HC (H) | Non-SUDD (N) | SUDD (S) | P-value | P-value | | |
| --- | --- | --- | --- | --- | --- | --- | --- |
|  | (n=34) | (n=75) | (n=33) | for F-test | H vs N | H vs S | N vs S |
| Blood test |  |  |  |  |  |  |  |
| hsCRP (mg/dL) | 0.095 (0.056) | 0.13 (0.12) | 0.43 (0.20) | <0.0001 | 0.37 | <0.0001 | <0.0001 |
| EAA (pg/mL) | 0.074 (0.051) | 0.13 (0.061) | 0.24 (0.075) | <0.0001 | <0.0001 | <0.0001 | <0.0001 |
| Fecal test |  |  |  |  |  |  |  |
| Calprotectin (μg/g) | 11 (7.2) | 16 (14) | 46 (29) | <0.0001 | 0.038 | <0.0001 | <0.0001 |

Data are shown as mean (standard deviation).

EAA, endotoxin activity assay; HC, healthy control; hsCRP, high-sensitivity C-reactive protein; SUDD, symptomatic uncomplicated diverticular disease

**Supplementary Table 11. Correlation of inflammatory markers with fecal BAs**

| Variables | r | |
| --- | --- | --- |
|  | Fecal calprotectin | Blood EAA |
| Total BA | 0.28 | 0.23 |
| Total primary BA | 0.23 | 0.15 |
| Total secondary BA | 0.28 | 0.24 |
| Total conj BA | 0.19 | 0.09 |
| Total unconj BA | 0.28 | 0.24 |

BA, bile acid; Conj, conjugated; r, correlation coefficient; Unconj, unconjugated; EAA, endotoxin activity assay

**Supplementary Table 12. Correlation of inflammatory markers with fecal unconjugated secondary BAs**

| Variables | | r | |
| --- | --- | --- | --- |
|  |  | Fecal calprotectin | Blood EAA |
| Unconj BA | |  |  |
|  | DCA | 0.20 | 0.15 |
|  | LCA | 0.25 | 0.30 |
|  | HDCA | 0.34 | 0.22 |
|  | UDCA | 0.43 | 0.32 |

BA, bile acid; DCA, deoxycholic acid; HDCA, hyodeoxycholic acid; LCA, lithocholic acid; r, correlation coefficient; UDCA, ursodeoxycholic acid; Unconj, unconjugated; EAA, endotoxin activity assay

**Supplementary Table 13. Correlation of NRS score, BSFS and DICA classification with fecal BAs**

| Variables | r | | |
| --- | --- | --- | --- |
|  | NRS | BSFS | DICA total point |
| Total BA | 0.16 | 0.29 | 0.18 |
| Total primary BA | 0.23 | 0.22 | 0.16 |
| Total secondary BA | 0.16 | 0.28 | 0.17 |
| Total conj BA | - | - | - |
| Total unconj BA | 0.16 | 0.29 | 0.18 |
| P/S | 0.16 | - | - |
| Total CA | 0.28 | 0.18 | 0.21 |
| Total CDCA | 0.15 | 0.21 | - |
| Total DCA | - | 0.21 | - |
| Total LCA | 0.16 | 0.26 | 0.20 |
| Total HDCA | 0.21 | 0.27 | - |
| Total UDCA | 0.37 | 0.33 | 0.23 |

r; correlation coefficient; NRS, numerical rating scale; BSFS, Bristol stool form scale; DICA, Diverticular Inflammation and Complication Assessment; BA, bile acid; CA, cholic acid; CDCA, chenodeoxycholic acid; DCA, deoxycholic acid; LCA, lithocholic acid; HDCA, hyodeoxycholic acid; UDCA, ursodeoxycholic acid; Conj, conjugated; Unconj, unconjugated; P/S, primary bile acid/secondary bile acid

# References

1. Ogawa Y, Imajo K, Honda Y, Kessoku T, Tomeno W, Kato S, et al. Palmitate-induced lipotoxicity is crucial for the pathogenesis of nonalcoholic fatty liver disease in cooperation with gut-derived endotoxin. Sci Rep (2018) 8:11365. doi: [10.1038/s41598-018-29735-6](https://doi.org/10.1038/s41598-018-29735-6).

2. Kato T, Honda Y, Kurita Y, Iwasaki A, Sato T, Kessoku T, et al. Lubiprostone improves intestinal permeability in humans, a novel therapy for the leaky gut: A prospective randomized pilot study in healthy volunteers. PLOS ONE (2017) 12:e0175626. doi: [10.1371/journal.pone.0175626](https://doi.org/10.1371/journal.pone.0175626).

3. Kessoku T, Imajo K, Kobayashi T, Ozaki A, Iwaki M, Honda Y, et al. Lubiprostone in patients with non-alcoholic fatty liver disease: a randomised, double-blind, placebo-controlled, phase 2a trial. Lancet Gastroenterol Hepatol (2020) 5:996-1007. doi: [10.1016/S2468-1253(20)30216-8](https://doi.org/10.1016/S2468-1253(20)30216-8).

4. Kasai Y, Kessoku T, Tanaka K, Yamamoto A, Takahashi K, Kobayashi T, et al. Association of serum and fecal bile acid patterns with liver fibrosis in biopsy-proven nonalcoholic fatty liver disease: an observational study. Clin Transl Gastroenterol (2022) 13:e00503. doi: [10.14309/ctg.0000000000000503](https://doi.org/10.14309/ctg.0000000000000503).

5. Tursi A, Brandimarte G, Di Mario F, Andreoli A, Annunziata ML, Astegiano M, et al. Development and validation of an endoscopic classification of diverticular disease of the colon: the DICA classification. Dig Dis (2015) 33:68-76. doi: [10.1159/000366039](https://doi.org/10.1159/000366039).

6. Tursi A, Brandimarte G, Di Mario F, Lanas A, Scarpignato C, Bafutto M, et al. The DICA endoscopic classification for diverticular disease of the colon shows a significant interobserver agreement among community endoscopists: an international study. J Gastrointestin Liver Dis (2019) 28(Suppl 4):39-44. doi: [10.15403/jgld-558](https://doi.org/10.15403/jgld-558).

7. Tursi A, Brandimarte G, Di Mario F, Elisei W, Picchio M, Allegretta L, et al. Prognostic performance of the ‘DICA’ endoscopic classification and the ‘CODA’ score in predicting clinical outcomes of diverticular disease: an international, multicentre, prospective cohort study. Gut (2022) 71:1350-8. doi: [10.1136/gutjnl-2021-325574](https://doi.org/10.1136/gutjnl-2021-325574)
